# Pyrazole Derivatives Induce Apoptosis via ROS Generation in the Triple Negative Breast Cancer Cells, MDA-MB-468

**DOI:** 10.31557/APJCP.2021.22.7.2079

**Published:** 2021-07

**Authors:** Maryam Ashourpour, Fatemeh Mostafavi-Hosseini, Mohsen Amini, Ebrahim Saeedian Moghadam, Faranak Kazerouni, Seyed Yousef Arman, Zahra Shahsavari

**Affiliations:** 1 *Department of Laboratory Medicine, Faculty of Paramedical Sciences, Shahid Beheshti University of Medical Sciences, Tehran, Iran. *; 2 *Department of Medicinal Chemistry, Faculty of Pharmacy and Drug Design & Development Research Center, The Institute of Pharmaceutical Sciences, Tehran University of Medical Sciences, Tehran, Iran. *; 3 *School of Chemistry, Australian Centre for Nano Medicine and the ARC Centre of Excellence in Convergent Bio-Nano Science and Technology, The University of New South Wales, Kensington, New South Wales, Australia. *; 4 *Department of Clinical Biochemistry, Faculty of Medicine, Shahid Beheshti University of Medical Sciences, Tehran, Iran. *

**Keywords:** Pyrazole derivatives, TNBC, Apoptosis, ROS

## Abstract

**Background:**

Triple-negative breast cancer accounts for approximately 15–20% of all breast carcinomas and is associated with earlier age of onset, aggressive clinical course, and dismal prognosis. A series of 1,3-diaryl-5-(3,4,5-trimethoxyphenyl)-4,5-dihydro-1 H-Pyrazole and 1,3-diaryl-5- (3,4,5-trimethoxyphenyl)- 1 H-Pyrazole were evaluated for their anticancer activity against MDA-MB-468, human triple negative breast cancer cell line.

**Methods:**

The cytotoxic effects of Pyrazole derivatives on the growth of MDA-MB-468 and AGO1522 were determined using MTT assay. Annexin-V-FITC and PI staining were performed to detect apoptosis and cell cycle distribution using Flow cytometry. The level of Reactive oxygen species (ROS) formation and caspase 3 activity were determined accordingly.

**Results:**

Pyrazole derivatives induced a dose and time-dependent cell toxicity in MDA-MB-468 compared with untreated cells. The results showed that 3-(4-methoxyphenyl)-1-(p-tolyl)-5-(3,4,5-trimethoxyphenyl)-4,5-dihydro-1H-Pyrazole (3f) was the most active compound with IC_50_ values 14.97 μM and 6.45 μM compared with Paclitaxel with IC_50_ values 49.90 μM and 25.19 μM, after 24 and 48 hours, respectively. Upon treatment with 14.97 μM of 3f after 24 h, the compound induced cell cycle arrest in S phase. 3f provoked apoptosis was accompanied by the elevated level of ROS and increased caspase 3 activity in MDA-MB-468 cells compared with untreated cells.

**Conclusion:**

The overall results of the present study provided evidence for the cytotoxicity of compound 3f against MDA-MB-468 cells in comparison to reference standard, Paclitaxel. It proves that compound 3f can trigger apoptosis through ROS production and caspase 3 activation. These bring supportive data for future investigations that will lead to their use in cancer therapy.

## Introduction

Breast cancer is one of the most common cancers among women and is a major reason for cancer death worldwide (Rakha et al., 2010). According to the literature, the incidence of female breast cancer in various countries was accounted for up to 3.2 million new cases per year by 2050 (Tao et al., 2015). Basically, breast cancer is a heterogeneous complex of diseases which involve a broad spectrum of subtypes (Yersal and Barutca, 2014). By the way illustration, luminal A (ER + and/or PR+, HER-2- ), luminal B (ER + and/or PR+, HER-2+), HER2-enriched (ER-, PR-, HER-2+), basal-like (ER-, PR-, HER-2- ) and normal-like are five biologically distinct intrinsic subtypes based on gene expression profiling classification (Liu et al., 2014; Jiang et al., 2019). The complex nature of breast cancer corresponds to distinct biological features results in various responses to different treatments. Since most basal-like tumors lack expression of hormone receptors (HR) and overexpression and/or amplification of HER2, triple-negative breast cancer (TNBC) refers to basal-like classification (Prat et al., 2013). Therefore, the design, development, and implementation of potential therapeutic molecules along with unveiling the myriad of questions arising from the interaction of TNBC biological cells/drugs has been an area of intensive research in cancer drug discovery (Sebastian et al., 2016). Among organic compounds, Pyrazoles have gained attention as promising scaffolds in the field of medicinal chemistry (Saueressig et al., 2018). Possessing unique properties like anticancer, antiangiogenic, and anti- metastatic action, Pyrazole derivatives deserve credit for their varied biological activities (Lehmann et al., 2017). Different Pyrazole derivatives including phenazone, metamizole, aminopyrine, phenylbutazone, sulfinpyrazone and oxyphenbutazone are readily available anti-inflammatory drugs (Prabhu et al., 2018). In this regard, Viagra , Celecoxib and Fipronil containing highly substituted Pyrazole ring have been commercialized as an effective ligand for estrogen receptors (Ananda et al., 2018). Sun et al., (2018) have reported the strong anti-tumor activity of these compounds for human lung cancer cell lines, A549. The anticancer efficiency of Pyrazole derivatives is reportedly connected to inhibition of several targets in cancer cells through interaction with topoisomerase II, EGFR, VEGF, HDAC, IGF-1R, Aurora-A kinase, cMet, Tubulin, mTOR , B-raf, CDKs, PI3K, JAK2, ALK, among others (Alam et al., 2016). Despite the fact that investigation pertinent to the antitumor activity of Pyrazole derivatives does not exceed two decades, the effectiveness of these compounds in the treatment of various cancers has been reported by many researchers. For instance, ruxolitinib (a selective inhibitor of JAK1 and JAK2), crizotinib (a c-Met and ALK inhibitor) and AT7519 (an inhibitor of cyclin-dependent kinases) have been reported for treatment of myelofibrosis, non-small cell lung carcinoma and refractory solid tumors respectively (Nitulescu et al., 2015). Based on published data, Pyrazoles induce cytotoxicity to human cancer cells through apoptosis pathway. The present work aims to evaluate the anticancer properties of Pyrazole derivatives against MDA-MB-468 triple negative breast cancer cell line.

## Materials and Methods

*Materials*

We used all chemicals as analytical grades without any further purification. Dulbecco’s modified Eagle’s medium/nutrient F-12 Ham (DMEM/Ham’s F12), Dulbecco’s Modified Eagle’s Medium-low glucose (DMEM/Low Glucose), Fetal bovine serum (FBS), Penicillin-streptomycin (P/S), phosphate-buffered saline (PBS) and 0.25 % trypsin–Ethylenediaminetetraacetic acid (EDTA), Dimethyl sulfoxide (DMSO) were obtained from Gibco (Grand Island ,NY,USA); 3-(4,5-dimethylthiazol-2-yl)-2,5-diphenyltetrazolium bromide (MTT), Fluorescent probe 2′,7′dichlorofluorescin diacetate (DCFH-DA) and Propidium iodide (PI) were obtained from Sigma-Aldrich (Munich, Germany). Annexin V-FITC apoptosis detection kit and Caspase-3/CPP32 Fluorometric Assay Kit were obtained from BioVision Research Products (Mountain View, CA, USA). The human breast cancer cell line, MDA-MB-468 and the normal human fibroblast cell line, AGO1522 were obtained from the Iranian Biological Resource Center (IBRC, Tehran, Iran).

*Cell culture*

The MDA-MB-468 cells were maintained in DMEM/Ham’s F12 supplemented with L-glutamine, with 10% fetal bovine serum and 1% penicillin/streptomycin. The AGO1522 cell line was cultured in DMEM/Low Glucose, with 10% fetal bovine serum and 1% penicillin/ streptomycin. Cells were incubated at 37°C, 5% CO2 and every 2-3 days at the confluency of 80%, the cells were harvested from the culture flask by Trypsin-EDTA.

*Cell viability assay*

Cell viability was measured with the MTT assay. The reduction of the tetrazolium salt, to a blue formazan crystal is facilitated by mitochondrial dehydrogenase and NADPH-dependent cellular oxidoreductase enzymes (Abel and Baird, 2018). In the assay, an aliquot of 7.5 × 10^3^ cells was seeded on the 96-well plates and allowed to attach overnight. Then, 11 Pyrazole derivatives (as shown in Table 1 and Figure S1) were added to the desired well with the final concentration of 3, 6.25, 12.5, 25, 50, 100 μM. After pre-treating cells for 24 and 48h, MTT (5 mg/ml in PBS) was added to each well and the cells were incubated for another 4 h at 37°C. In order to dissolve blue-colored formazan crystals, the entire medium was aspirated after the incubation period, and DMSO was added to the wells. An ELISA plate reader (BioTek Instruments, Inc., USA) was used to quantify the formazan concentration (with the wavelengths of 570 nm). All of the experiments were carried out on three replicates to ensure the measurement repeatability and the IC_50_ values were also calculated using GraphPad Prism software.

*Detection of apoptosis using annexin V/PI staining*

Annexin V-FITC/PI Apoptosis Detection Kit was employed to detect apoptosis. To assess the number of apoptotic cells, an aliquot of MDA-MB-468 cells was adjusted to obtain 2 × 10^5^ cells per well in six-well plates followed by incubation for an overnight. After incubation cells on the surface for an appropriate time, compound 3f was added to the wells to obtain the final concentration of 14.7μM in wells for 24 h. Cells were then harvested from the culture wells by aspirating media and washing with ice-cold PBS. Once the cells have detached, resuspended cells were pipetted up and down thoroughly followed by transferring to a sterilized 5 ml centrifuge tube. After 15min, 400 µl of the binding buffer was added to the cell suspension. Then annexin V-FITC and PI staining solution were added to the tubes. The cell suspension was then placed in a dark place (10 min at room temperature). A flow cytometer (BD Biosciences, San Jose, CA) was used to analyze the cells. The analysis of data was made using FlowJo software.

*Cell cycle analysis*

Cell cycle analysis was conducted through taking advantage of PI staining and flow cytometry analysis. To evaluate cellular DNA levels, the cells stained with PI were subjected to ﬂow cytometric analyses. In this assay, six-well plates were loaded with 2 × 10^5^ MDA-MB-468 cells / well. After incubation of the samples at 37°C for 24 h, cells were treated with 3f at concentrations of 14.7 μM followed 24 h incubation. Then, the effect of the drug became evident and cells collected by rinsing with PBS (two times), immobilizing with 70% ice-cold ethanol (for 3 h), and washing with PBS (two times). To resuspend the cells a mixture of 0.25 ml PBS containing 5 μl of 10 mg/ml RNase A and Triton X-100 (0.1%) including 10μl of PI (50 μg/ml) was used and incubation was performed at 37°C for 30 min in a dark place. The assessment of stain cells was carried out employing a FACS Calibur flow cytometer (BD Biosciences, San Jose, CA). The apoptotic rate of the cell population was measured by calculating the percentage of cells with sub-G1 DNA content. 

*Detection of intracellular reactive oxygen species (ROS)*

The level of intracellular ROS corresponded to the MDA-MB-468 cells in response to the 3f compound was monitored by using DCFH-DA. Briefly, 7.5 × 10^3 ^MDA-MB-468 cells were seeded per well of 96-wells plate and allowed to attach to the bottom of the wells for an overnight followed by treatment with 3f compound at a concentration of 14.7 μM. After incubation of cells for 24 h, PBS was used for rinsing and then cells incubated in the presence of DCFH-DA for 30 min at 37°C. Next, to remove the extracellular DCFH-DA, cells were washed three times with prechilled PBS. Finally, the fluorescent of collected cells was analyzed by using a fluorescent microplate reader (BioTek Synergy HT, Winooski, VT, USA).

*Determination of caspase-3 activity*

From a mechanistic point of view, caspase 3 as a member of the cysteine-aspartic protease family is essential for a variety of biological activities, including apoptosis in mammalian cells (Skiba et al., 2019). To confirm the drug triggers apoptosis, the level of 7-amino-4-trifluoromethyl-coumarin (AFC) from DEVD-AFC, a marker of caspase 3 activation, was measured using Caspase-3/CPP32 Fluorometric Assay Kit (BioVision Research Products, Mountain View, CA, USA). Briefly, MDA-MB-468 cells (a density of 7.5 × 10^3^ per well in 96-wells plate) were treated with 14.97 μM 3f for 24 h. The following day, cells were collected, washed two times in PBS and lysed in lysis buffer (for 10 min on ice). The concentration of protein was assessed and 50 µL of supernatant containing 50-200 µg protein was added to the 50 µL of 2x reaction buffer and 5 μl of DEVD-AFC substrate (1 mM), in a 96-wells plate. The mixture was then left at 37°C for 2 hours followed by monitoring the fluorescence intensity using a fluorescent microplate reader (BioTek Synergy HT, Winooski, VT, USA).

*Statistical analysis*

All experiments were carried out on three replicates and the data including uncertainties were presented as Mean ± SD. All data analysis was performed using GraphPad Prism software 6 (CA, USA). A one-way analysis of variance followed by Dunnett’s test was used to compare the data between the groups. A value of p<0.05 was considered significant.

## Results

*3f inhibits MDA-MB-468 cells proliferation in a dose- and time-dependent manner*

The cytotoxic effect of compounds on MDA-MB-468 and AGO1522 were studied by in vitro MTT assay with Paclitaxel as the standard. Regardless of concentration, a drop in cell viability occurred in a time-dependent manner for all compounds indicating the effectiveness of drugs compared with the untreated control sample (Table 2). The observation would suggest the superior activity offered by 3f compound among the other synthesized compounds on the MDA-MB-468 breast cancer cell line. The IC_50_ value of MDA-MB-468 correspond to the 3f compound after 24, and 48 h treatment were recorded 14.97 μM and 6.4 μM while with those of control drug Paclitaxel were 49.9 μ and 25.19 μM, respectively. Further inspection of the results revealed 3f compound not only induces the maximum inhibitory growth on breast cancer cells (MDA-MB-468) but also the minimum toxicity on normal fibroblast cell line (AGO1522) indicating the effectiveness of this compound on inhibiting human triple negative breast cancer cells.

*Compound 3f induces apoptosis in MDA-MB-468 breast cancer cells*

**Figure 1:**
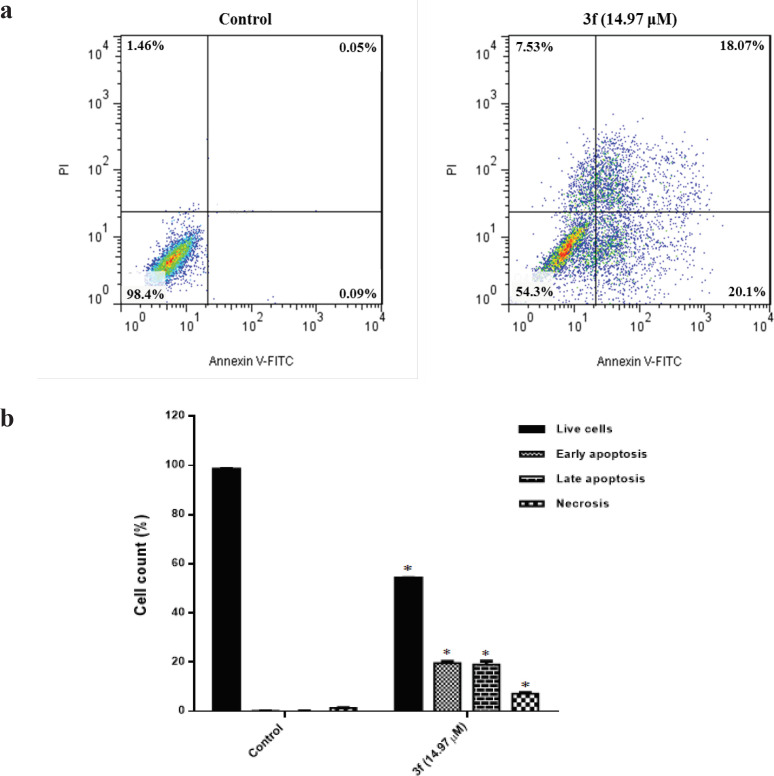
(a), The flow cytometry analysis of MDA-MB-468 cell line after 24 h treatment with 3f compound; (b), The percentages of live cells, necrosis, early and late apoptosis

To provide a better understanding of the cytotoxicity mechanism behind the 3f compound, we used flow cytometry following annexin V-FITC/PI double staining. From the data presented in Figure 1a, the apoptosis value corresponds to MDA-MB-468 cells in the presence of compound 3f (14.97 µM) after 24 h compared with the control sample (untreated cells) was calculated 38%. In addition, as time elapsed up to 24 h treatment with 3f compound, the intensities related to necrotic cells (stained with PI) and late apoptotic cells (stained with annexin V and PI) encountered a significant rise. Figure 1b reveals that the early apoptotic stage of MDA-MB-468 cells (stained with annexin V) in response to 3f compound increased significantly compared to the control sample. Accordingly, the finding could enable us to believe that apoptosis is the mechanism by which 3f compound triggers cytotoxicity in breast cancer cells.

*3f leads to the accumulation of the MDA-MB-468 cells in the subG1 phase of the cell cycle*

**Figure 2:**
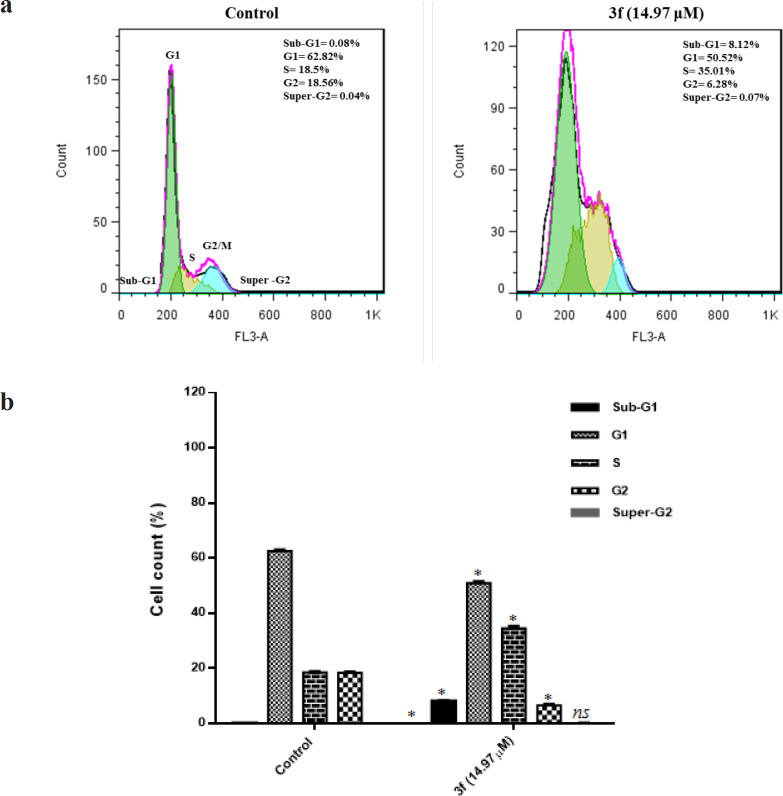
(a) The histograms of DNA content for MDA-MB-468 cell line treated with 3f for 24 h. (b) the percentage of cell cycle distribution after 24 h treatment with 3f compound

In the recent decade, an increasing interest toward applying cell cycle as a new approach for cancer therapy could be spotted among researchers. For instance, it is well established that there is a correlation between cell cycle progression and inhibition of cell proliferation and apoptosis in cancer cells (Cankara Pirol et al., 2014). We performed Flow cytometric analyses in combination with DNA staining using PI to study cells distribution in the different phases of the cell cycle. Our results revealed that treatment of triple negative breast cancer cells with 3f compound results in an increasing number of the cells at G0/G1 phase and S-phase. From Figure 2a, by introducing 14.97 μM of 3f compound to the culture media of MDA-MB-468 for 24 h, the fraction of cells at the Go/G1 and S phase met a rise from 0.08 % to 8.12 % and 18.5 % to 35 % respectively. Calculation of cell cycle distribution pertinent to MDA-MB-468 cells revealed that treatment of cells with 3f compound for 24 h leads to an increase in cell death by apoptosis. Engaging the reaction process in Figure 2b, 3f could be considered as an effective inducer of apoptosis in triple negative breast cancer.

*ROS generation plays critical roles in 3f-induced cell death*

**Figure 3:**
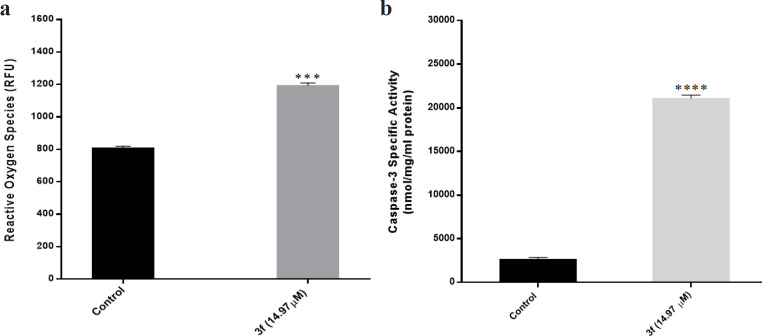
(a) The ROS generation after 24 h treatment with 3f compound. (b) The caspase-3 activation in MDA-MB-468 cells after 24 h treatment with 3f compound. The measurement was performed by an enzymatic assay. An increase in caspase-3 activation can be observed in response to 3f treatment

To study the role of reactive oxygen species in inducing apoptosis by compound 3f, DCFH-DA, a fluorescent probe, was used to quantify the level of ROS production. Accordingly, 14.97 μM of 3f compound was added to the MDA-MB-468 breast cancer cells and then after 24 h, the ROS level was measured by fluorimetry. From Figure 3a, an ascending trend pertinent to the intracellular ROS can be observed in MDA-MB-468 cells in the presence of 3f compound. This behavior may reflect that the induction of apoptosis in MDA-MB-468 triple negative breast cancer cells by 3f compound is linked to ROS production. 

*Caspase-3 activities in compound 3f mediated apoptosis*

The fluorimetric assay kit was applied to measure the activity of caspase-3 as a major protease in apoptosis signaling. The representative caspase-3 activity in MDA-MB-468 cells in the presence and absence of 3f compound has been depicted in Figure 3b. In the presence of 3f compound, the caspase activity significantly increased. 

**Table 1:** Pyrazole Derivatives Characteristics

		Molecular weight (g/mol)	R1	R2
3a	1,3-diphenyl-5-(3,4,5-trimethoxyphenyl)-4,5-dihydro-1H-Pyrazole	388.46	H	H
3b	3-phenyl-1-(p-tolyl)-5-(3,4,5-trimethoxyphenyl)-4,5-dihydro-1H-Pyrazole	402.49	H	Me
3c	1-(4-chlorophenyl)-3-phenyl-5-(3,4,5-trimethoxyphenyl)-4,5-dihydro-1H-Pyrazole	422.9	H	Cl
3d	1-(4-fluorophenyl)-3-phenyl-5-(3,4,5-trimethoxyphenyl)-4,5-dihydro-1H-Pyrazole	406.45	H	F
3e	3-(4-methoxyphenyl)-1-phenyl-5-(3,4,5-trimethoxyphenyl)-4,5-dihydro-1H-Pyrazole	418.49	OMe	H
3f	3-(4-methoxyphenyl)-1-(p-tolyl)-5-(3,4,5-trimethoxyphenyl)-4,5-dihydro-1H-Pyrazole	432.52	OMe	Me
3g	1-(4-chlorophenyl)-3-(4-methoxyphenyl)-5-(3,4,5-trimethoxyphenyl)-4,5-dihydro-1HPyrazole	452.93	OMe	Cl
4a	1,3-diphenyl-5-(3,4,5-trimethoxyphenyl)-1H-Pyrazole	386.45	H	H
4b	3-(4-methoxyphenyl)-1-(p-tolyl)-5-(3,4,5-trimethoxyphenyl)-1H-Pyrazole	416.47	OMe	Me
4c	1-(4-chlorophenyl)-3-(4-methoxyphenyl)-5-(3,4,5-trimethoxyphenyl)-1H-Pyrazole	450.91	OMe	Cl
4d	1-(4-fluorophenyl)-3-(4-methoxyphenyl)-5-(3,4,5-trimethoxyphenyl)-1H-Pyrazole	434.46	OMe	F

**Table 2:** IC_50_ Values of Novel Pyrazol Compounds and Paclitaxel after 24 and 48 h Treatment in MDA-MB-468 and AGO-1522 Cells

	MDA-MB-468		AGO1522	
	24 hours	48 hours	24 hours	48 hours
3a	*25.00 ± 0.92	6.41 ± 1.10	28.80 ± 0.95	19.87 ± 0.83
3b	19.20 ± 0.97	9.29 ± 0.67	29.82 ± 1.02	19.78 ± 0.96
3c	58.61 ± 0.82	19.87 ± 1.12	24.10 ± 0.65	17.65 ± 0.93
3d	32.58 ± 1.22	15.25 ± 0.90	42.15 ± 1.27	35.57 ± 1.32
3e	21.31 ± 0.85	10.81 ± 0.93	42.79 ± 0.98	19.89 ± 0.99
3f	14.97 ± 1.09	6.45 ± .84	28.74 ± 1.20	20.33 ± 0.83
3g	25.83 ± 0.91	8.50 ± 0.89	43.30 ± 0.96	33.97 ± 1.92
4a	19.35 ± 1.14	11.03 ± 0.88	31.19 ± 1.24	14.48 ± 0.97
4b	58.20 ± 1.35	11.95 ± 0.94	34.56 ± .89	27.03 ± 0.79
4c	28.76 ± 0.98	12.42 ± 0.79	25.19 ± 0.88	20.95 ± 0.92
4d	58.58 ± 1.32	24.46 ± 1.17	34.41 ± 1.31	27.34 ± 0.94
Paclitaxel	49.90 ± 0.97	25.19 ± 1.27	42.93 ± 1.42	22.59 ± 0.91

*Data shown are mean ± SD of triplicate samples

## Discussion

Among many different types of breast cancer, TNBC is the most aggressive one with a higher frequency in minority populations (Kastrati et al., 2010). Even though chemotherapy is still one of the efficient ways to target cancerous tumors, the performance of these anticancer agents is limited to the selectivity of the drug by cancer cells and other tumor-related factors including the development of drug resistance over time (Atmaca et al., 2017). The rise of concern about the drawbacks associated with common drugs points up the need for more research toward a new generation of anticancer agents (Tessmann et al., 2017). On the basis of published data, heterocyclic compounds have been widely utilized in modern therapeutics (Saleh et al., 2016). Possessing a variety of biological properties including anticancer, antioxidant, anti-inﬂammatory, antibacterial, antimicrobial, antidepressant, antiangiogenic, anti-inﬂuenza, and analgesic activities, Pyrazole derivatives have been documented as a promising compound for specific application in drug discovery (Inceler et al., 2013; Li and Zhao, 2014). In this research work, the impact of Pyrazole derivatives on the human triple negative breast cancer cell line was investigated. Our results revealed the anti-proliferation response of cancer cells to drug in a dose- and time-dependent manner. We assessed several synthesized Pyrazole derivatives and 3f exhibited a significant inhibitory response on cancer cells. The IC_50_ value obtained after 24 h treatment for MDA-MB-468 cancer cell and AGO1522 normal fibroblast cell line were recorded 14.97 and 28.74 μM respectively, indicating the effectiveness of 3f compound on inhibiting the growth of human triple negative breast cancer cells. Our results are in agreement with the literature that reported the apoptosis effect of Pyrazole derivatives on cancer cells (Inceler et al., 2013; Sebastian et al., 2016; Ananda et al., 2018; Nossier et al., 2018). The regulated destruction of a cell or apoptosis is a vital physiological process in elimination and suppression of cancer tumors (Agarwal et al., 2018). Due to acquiring resistance by tumors, chemotherapy inducing apoptosis have been investigated through the years in the hope of finding a proper compound to overcome apoptosis resistance (Shahsavari et al., 2015; Fakai et al., 2019). In this research work, we used the Annexin-V/FITC and PI staining to understand whether apoptosis is involved in mechanism behind interaction of 3f compound and MDA-MB-468 cells. The results disclosed signiﬁcant level of cell death by a major contribution of the apoptotic pathway. Pyrazole derivatives as an inducer of apoptosis in cancer cells have already been reported. Demetrio Raffa et al., (2019) studied a series of Pyrazole derivatives on a human lung carcinoma cell line and they revealed the apoptotic cell death by activating TRAIL death receptors followed by caspase-8 activation (extrinsic pathway) or changes in p53 activation (intrinsic pathway). Another study (Czarnomysy et al., 2018) investigated the complexation of Pyrazole derivatives with platinum to enhance their therapeutic effect. The results associated with the interaction of two breast cancer cell lines and Novel Pyrazole platinum (II) complexes disclosed a higher percentage of DNA fragmentation indicating apoptosis via indirect DNA damage. Uncontrolled cell proliferation is the cancer’s common traits as a result of mutated genes that directly control cell cycle (Kong et al., 2016). It is well established that the cell cycle progression inhibition is an efficient approach to hinder cancer tumor progression (Liu et al., 2016). There has been a great deal of research into potential benefits of targeting cell cycle including a possible opportunity to deal with acquiring drug resistance (Prasedya et al., 2016). In the present work, we observed a drastic rise of sub-G0/G1 DNA content in cells indicating significant increase of apoptotic cell death. A similar result was reported by Mohamed et al., (2014) who revealed a rise of G0/G1 phase for MCF-7 cells in response to the treatment with a synthetic compound containing Pyrazole ring. In an effort to introduce a new anticancer agent for bladder cancer, a series of pyrazoline derivatives was utilized on 5647 and T24 cells as two human bladder cancer cell line. The evidence provided revealed an inhibited cell cycle progression of 5637 cells in the presence of 1-thiocarbamoyl-3,5-diphenyl-4,5-dihydro-1H-Pyrazole (2a) and 1-thiocarbamoyl-5-(4-chlorophenyl)-3-phenyl-4,5-dihydro-1H-Pyrazole (2c). In addition, a dramatic increase of sub G0/G1 phase was observed which linked to the apoptosis (Tessmann et al., 2017). 

According to the literature (Ji et al., 2013), a high amount of ROS may results in the oxidative destruction of cellular protein, lipids, nucleic acids and finally triggers the cellular death pathway through apoptosis or necrosis. Mitochondrial apoptotic pathway through ROS production is a ubiquitous feature of chemotherapeutic agents (Saleh et al., 2016). Therefore, novel chemotherapeutic agents regulating the accumulation of intracellular ROS may be considered as a promising approach to eliminate tumor growth (Tsai et al., 2017). Our study disclosed the selective growth inhibition of breast cancer cells, MDA-MB-468 corresponded to the 3f compound is associated with apoptosis pathway through ROS generation. Our results are in agreement with the literature (Xiong et al., 2019) that reported a novel thiazolyl substituted bis-pyrazole oxime compound selectively inhibited proliferation of colorectal cancer HCT116 cells by inducing apoptosis pathway through the promotion of intracellular ROS level. The therapeutic impact of a Pyrazole compound on angiogenesis in human umbilical vein endothelial cells (HUVECs) was studied by Zhang et al., (2011). They showed the Pyrazole compound induced angiogenesis by a significant increase of NO and ROS production through ROS-HIF-1α-VEGF and NO signaling pathways. To put it simply, the levels of HIF-1α and VEGF in the presence of Pyrazole compound enhanced in a ROS dependent manner. 

Cysteine proteases namely caspases play a vital role in the programmed cell death affecting the coordinated cascade for degrading cellular components (Cao et al., 2015). Caspase-3, as a member of the cysteine-aspartate-specific protease family, is a ubiquitous protein in mammalian cells contributing to apoptosis significantly (Zhao et al., 2014). The caspase-3 causes apoptosis pathway can be activated by a myriad of stimuli including chemotherapeutic compounds (Lepiarczyk et al., 2015). Herein, we evaluated whether caspase-3 activation plays a role in the apoptosis triggered by 3f compound or not. We observed a significant rise of caspase-3 enzymatic activity in the presence of 3f compound indicating this Pyrazole compound induced apoptosis in MDA-MB-468 cells. The evidence provided could lead us to believe that the caspase-dependent pathway is the significant mechanism which accelerates the apoptosis process induced by 3f compound in MDA-MB-468 cells. The cell growth attenuation, activation of caspase-dependent pathway, and fragmented DNA in HL-60RG leukemia cells induced by a Pyrazole compound was indicated by Nagahara and Nagahara, (2017). More recently, Harras et al., (2018) reported an excellent anti-cancer activity of a Pyrazole derivative against HCT116, UO-31, and HepG2 cells. They suggested a better cytotoxic activity of the Pyrazole compound in comparison with Sorafenib (a reference drug) through activation of caspases-3 dependent pathway. 

In this research work the cytotoxicity of a series of Pyrazole derivatives including 3-(4-methoxyphenyl)-1-(p-tolyl)-5-(3,4,5-trimethoxyphenyl)-4,5-dihydro-1H-Pyrazole namely 3f on MDA-MB-468 cells were proved. Evidence provided revealed the apoptosis activity of 3f compound on MDA-MB-468 triple negative breast cancer cells through ROS generation and caspase 3 signaling pathway. Therefore, it seems realistic to anticipate that Pyrazole derivatives might be used in the future and become a chemotherapeutic approach in cancer biology.

## Author Contribution Statement

Dr. Zahra shahsavari and Dr. Faranak Kazerouni jointly worked on the design and implementation of project and preparation of the manuscript. Maryam Ashourpour and Fatemeh Mostafavi-Hosseini jointly worked on the project implementation, statistical analysis and preparation of the manuscript as MSc. Students. Mohsen Amini and Ebrahim Saeedian Moghadam synthesized the Pyrazole derivative. Dr. Yousef Arman contributed the manuscript writing and editing. 
